# Evaluating the incidence of bacteriuria in female patients before and after implementation of external urinary collection devices

**DOI:** 10.1017/ash.2022.30

**Published:** 2022-03-17

**Authors:** Mandee M. Noval, Surbhi Leekha, Michael Armahizer, Abigale Celotto, Meghna Bhatt, Hyunuk Seung, Kimberly Claeys

**Affiliations:** 1 Department of Pharmacy, University of Maryland Medical Center, Baltimore, Maryland; 2 Department of Epidemiology & Public Health, University of Maryland School of Medicine, Baltimore, Maryland; 3 Department of Nursing, University of Maryland Medical Center, Baltimore, Maryland; 4 Department of Pharmacy, University of Maryland School of Pharmacy, Baltimore, Maryland

## Abstract

External urinary collection devices (ECDs) are increasingly used in female patients, however, their impact on bacteriuria and antimicrobial use is unclear. Comparing the periods before and after the implementation of an ECD use policy, we found an overall decrease in bacteriuria but no significant decrease in trend of monthly rates. Antimicrobial use for genitourinary indications did not change.

Internal urinary catheter (IUC) placement occurs in ∼25% of hospitalized patients and is associated with development of bacteriuria at a rate of 3%–7% per day.^1^ Catheter-associated urinary tract infections (CAUTIs) remain among the most common hospital-associated infections, and even in the absence of symptoms or signs of infection, asymptomatic catheter-associated bacteriuria is frequently inappropriately treated with antimicrobial therapy.^2^

Alternatives to IUCs, such as external collection devices (ECDs), are increasingly being used to reduce the incidence of CAUTI.^3,4^ ECDs have been used in men for years, with benefits of increased patient comfort and reduced catheter-associated complications.^2,3^ Although less studied, such devices have gained popularity in female patients.^5^ Use of ECDs in female ICU patients has been associated with decline in IUC use and a reduction in the CAUTI rate.^6,7^ Studies of the impact of female ECDs on the incidence of bacteriuria, which often leads to unnecessary antimicrobial use, are scarce.^8^ We hypothesized that increased ECD use, and decreased IUC use, in female ICU patients would lead to a decline in bacteriuria, decreasing both CAUTI diagnoses and antimicrobial use for urinary indications.

## Methods

In this quasi-experimental study, we evaluated ECD use in female ICU patients at the University of Maryland Medical Center (UMMC). We compared patients in the period before ECD use was implemented (December 2015–May 2017) to those in the period after ECD use was implemented (December 2017–June 2019). The study included adult female patients who had either an IUC or ECD placed during these periods in the medical, surgical, neurocritical, or cardiac surgery ICUs.

At UMMC, the PureWick Female ECD (Becton Dickinson, Franklin Lakes, NJ) use began in November 2017. Thereafter, female patients were eligible for an ECD if they met any of the following criteria: incontinence where movement for urination was not possible, stage III–IV pressure ulcers that could not be kept clear of incontinence, accurate daily measurement of urine volume was required, or improved comfort for palliative patients. IUCs were used for strict (hourly) measurement of urine output, urinary retention, and urinary tract injury.

Patient data were extracted from the institutional validated relational databases containing administrative, pharmacy, laboratory, and clinical data. These data included demographics, *International Classification of Diseases Tenth Revision* (ICD-10) diagnosis codes, urinalysis and urine culture results, and days of IUC or ECD use. An Elixhauser score was calculated using ICD-10 codes. All antimicrobial orders with a genitourinary urinary tract infection or “GU-UTI” indication were used to calculate urinary antimicrobial days of therapy (DOT). CAUTI cases meeting the NHSN surveillance definition were obtained from infection prevention databases.

The primary objective of our study was to characterize the incidence of bacteriuria in female ICU patients before versus after ECD use was implemented, measured as the number of positive urine cultures (presence of ≥10^5^ cfu/mL of bacteria or yeast) per 1,000 patient days (PD). Pre- versus post-ECD rates of urine cultures “ordered” and “performed” were measured separately because UMMC utilizes conditional urine culturing on urine WBC counts, so not all ordered cultures were performed. Secondary objectives were to evaluate the monthly rate of IUC use per 1,000 PD, incidence of CAUTI per 1,000 device days, and antimicrobial use for GU-UTI indication. Antimicrobial use was measured as both proportion and DOT normalized to 1,000 PD, before versus after ECD use was implemented.

Bivariate comparisons between the pre– and post–ECD-implementation groups were conducted using the χ^2^ or the Fisher exact test for categorical variables and the Mann-Whitney *U* test for continuous variables. Segmented regression analysis of interrupted time series (ITS) was used to evaluate changes between the pre- and post-ECD groups using a generalized linear model with Poisson or negative binomial distribution, as appropriate. Autocorrelation and seasonality were assessed using the Dubin-Watson test and the Dickey-Fuller test, respectively. A *P* value <.05 was considered statistically significant. All analyses were performed with SAS version 9.4 software (SAS Institute, Cary, NC).

## Results

Overall, 4,640 female patient–ICU encounters occurred during the study period; 2,201 before the ECD and 2,439 after the ECD, representing 18,779 PD and 21,098 PD, respectively. We detected no significant differences between groups with respect to most comorbidities; history of renal dysfunction was numerically higher in the post-ECD group. All patients in the pre-ECD group had an IUC, compared to 68.4% in the post-ECD group.

**Fig. 1.**
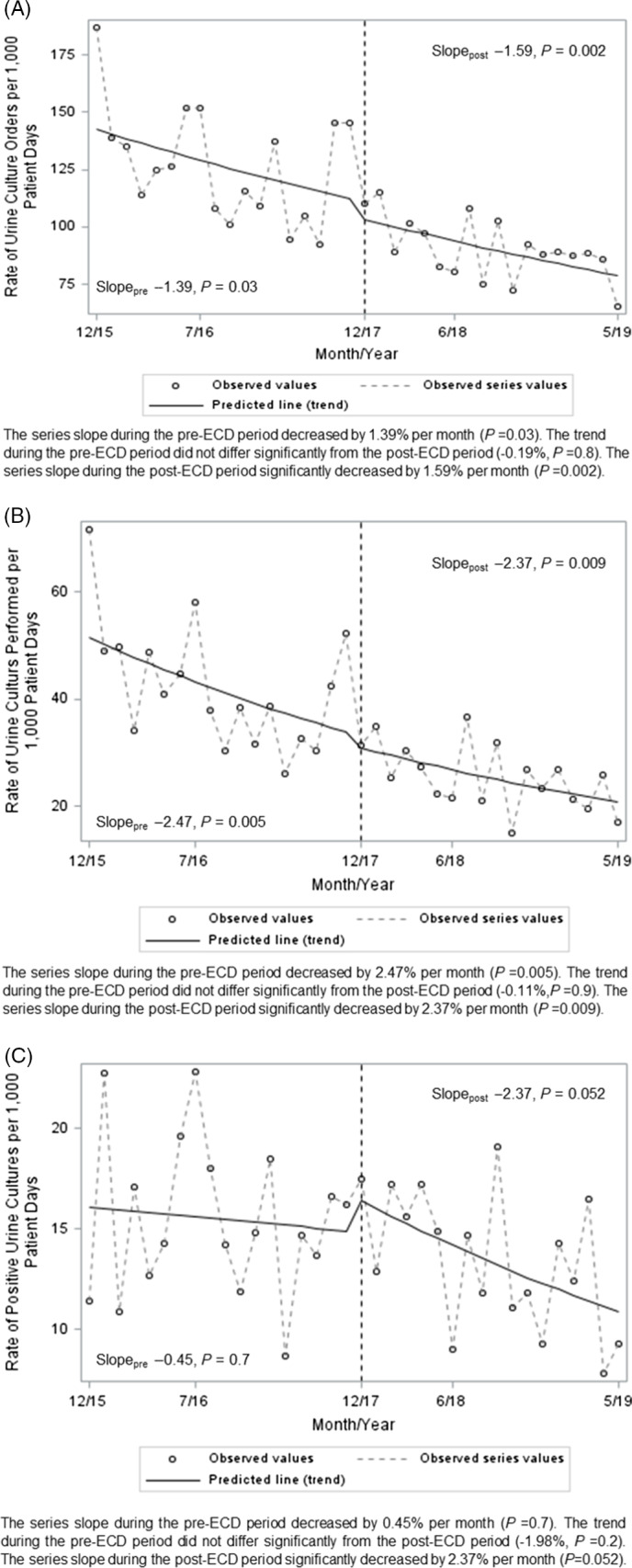
Segmented regression of interrupted time series (ITS) analysis was used to evaluate the changes of trends in the rates of (A) urine cultures ordered, (B) urine cultures performed, and (C) positive urine cultures per 1,000 patient days in the period before ECD use was implemented (December 2015–May 2017) versus after ECD use was implemented (December 2017– June 2019).

In ITS analysis, the mean rate of urine cultures ordered and performed showed significant monthly decreases both before and after the ECD use policy was implemented, but the rate of decrease did not change relative to ECD implementation (Fig. 1A and 1B). The overall incidence of bacteriuria was significantly lower after ECD implementation (28 positive urine cultures per 1,000 PD) compared to the period before ECD implementation (38 positive urine cultures per 1,000 PD; *P* = .004). Through ITS, pre-ECD rates showed a decrease of 0.45% per month (*P* = .70) and post-ECD rates decreased by 2.4% per month (*P* = .052). The change in slope (rate of decrease) from the pre-ECD period to the post-ECD period was not significant (−1.98%; *P* = .20) (Fig. 1C).

**Fig. 2.**
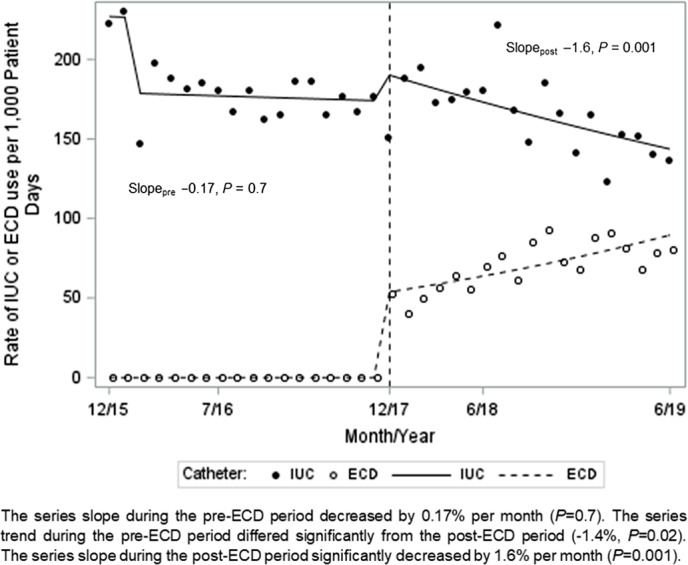
Segmented regression of interrupted time series (ITS) analysis to evaluate the changes of trend in the rate of IUC use per 1,000 patient days in the period before ECD use was implemented (December 2015–May 2017) versus the after ECD use was implemented (December 2017– June 2019).

In our ITS analysis, monthly IUC use did not change before ECD implementation, but it decreased significantly after ECD implementation by 1.6% per month (*P* = .001), with a significant pre- to post-ECD slope change of −1.4% (*P* = .02) (Fig. 2). The incidence of reportable CAUTIs declined post-ECD implementation: 4.8 per 1,000 IUC days versus 2.2 per 1,000 IUC days (*P* = .07) and 0.85 per 1,000 PD versus 0.33 per 1,000 PD (*P* = .036). Antimicrobial prescribing for GU–UTI indication (Supplementary Table 1) remained similar between the groups (16.9% before the ECD vs 15.9% after the ECD; *P* = .70), as did DOTs (1.9 per 1,000 PD before the ECD vs 1.8 DOT per 1,000 PD after the ECD; *P* = .70).

## Discussion

Following the implementation of the use of ECDs in female patients, the overall incidence of bacteriuria decreased, but the month-to-month rate and the change in this rate after the ECD policy was implemented were not significantly different. We observed similar trends with urine culture ordering. Therefore, although ECD use may have contributed to this decrease, factors outside ECD use, particularly longstanding and continuous efforts to decrease unnecessary urine culturing, may also be responsible for a downward secular trend in bacteriuria.

The observed overall reduction in bacteriuria is similar to prior studies of ECD use in male patients.^2,3^ A main driver of ECD use is to prevent CAUTIs by reducing IUC use. In our study, we observed a reduction in IUC use and lower CAUTI rates after the implementation of ECD use. Although CAUTI reduction was likely multifactorial, the role of ECDs reducing IUC use is supported by similar findings noted in previous studies in both female and male patients.^6,7^

We had further hypothesized that through reduction in bacteriuria, the implementation of ECD use would lead to a decline in antimicrobial use for UTIs; however, this was not observed. Inappropriate antimicrobial use in male patients with ECDs has previously been described, with upward of 32% of patients with ECD-associated asymptomatic bacteriuria receiving unnecessary antimicrobials.^1^ This discordance between decline in bacteriuria and antimicrobial use may be related to excessive use of antimicrobials for suspected sepsis in critically ill patients, where timely empiric antimicrobial therapy is stressed.^9^

This study had several limitations. We lacked a control group, and we were unable to account for other possible causes of decreases in bacteriuria between the pre-ECD and post-ECD periods. We were unable to evaluate the presence of true symptomatic UTIs. We only evaluated antimicrobials with GU-UTI indications to focus on antimicrobial prescriptions most likely to be causally related to urine culture results. However, patients in the ICU often initially present with nonspecific signs and symptoms of sepsis only later identified with a genitourinary source, and sepsis indication was not evaluated.

Implementation of ECD use was associated with overall reductions in IUCs and CAUTI. Although rates of bacteriuria declined overall, the direct impact of ECD use remains unclear. Additionally, we detected no difference in antimicrobial use for GU-UTI indications. Thus, an opportunity remains to improve diagnostic and antimicrobial stewardship practices related to catheter- and ECD-associated bacteriuria.

## Supplementary material

For supplementary material accompanying this paper visit https://doi.org/10.1017/ash.2022.30. click here to view supplementary material
