# Supplementary material for: Evaluating the incidence of bacteriuria in female patients before and after implementation of external urinary collection devices
Source: Antimicrob Steward Healthc Epidemiol. 2022 Mar 17;2(1):e44. doi: 10.1017/ash.2022.30 (PMC9614887; doi:10.1017/ash.2022.30)
Supplement: Supplementary file 1 [file S2732494X22000304sup001.docx]

**Supplemental Table: Antimicrobials for GU-UTI Indication**

|  | **Pre-ECD (n=712)** | **Post-ECD (n=789)** | **P-value** |
| --- | --- | --- | --- |
| **Antimicrobials**  Aminoglycoside  BL/BLI  Carbapenems  Cephalosporin  Fluconazole  Fluoroquinolone  PCN  TMP/SMX  Vancomycin  Other | 23 (3.2)  84 (11.8)  36 (5.1)  297 (41.7)  3 (0.4)  185 (26)  7 (1)  40 (5.6)  29 (4.1)  8 (1.1) | 41 (5.2)  84 (10.7)  61 (7.7)  364 (46.1)  30 (3.8)  92 (11.7)  8 (1)  37 (4.7)  18 (2.3)  54 (6.8) | 0.07  0.51  0.04  0.09  <0.001  <0.001  1.00  0.48  0.05  <0.001 |

BL/BLI = beta-lactam/beta-lactamase inhibitor; PCN = penicillin (ampicillin, amoxicillin)
